# Cervical Intraepithelial Neoplasia: Analyzing the Disease Present Exclusively in the Endocervical Canal

**DOI:** 10.1055/s-0042-1743102

**Published:** 2022-03-11

**Authors:** Fernanda Villar Fonseca, Maria Victória Gutierrez Cordeiro, Ariadne Cristine Pozza, Carlos Afonso Maestri

**Affiliations:** 1 Department of Gynecology and Obstetrics, Universidade Positivo, Curitiba, PR, Brazil

**Keywords:** CIN, oncotic cytology, cervical intraepithelial neoplasia, NIC, citologia oncótica, neoplasia intraepiteliais cervicais

## Abstract

**Objective** To evaluate the role of cervical cytology (Pap smear) in the diagnosis of cervical intraepithelial neoplasia 2 or greater (CIN2 + ), presented exclusively in the endocervical canal, the clinical-epidemiological characteristics of this lesion, the necessary length of canal to be removed to treat, and the rate of invasive lesion hidden in the endocervical canal.

**Methods** Cross-sectional study, by database analysis, of patients with abnormal cytology (high-grade squamous intraepithelial lesion [HSIL]), without visible colposcopy lesion, submitted to loop electrosurgical procedure (LEEP) to evaluate the association of cytology results with the histological product of the conization, to identify the epidemiological characteristics of endocervical lesion and clinical evolution, using a *p* -value < 0.05 and 95% CI.

**Results** In 444 cases, the Pap smear sensitivity for CIN2+ diagnosis was 75% (95% CI: 69.8–79.7), specificity was 40% (95% CI: 30.2–49.5), and the prevalence rate of histological lesion was 73% (95% CI: 70.1–78.7). There was a higher prevalence of CIN2+ in women over 42 years old and invasive cancer in those over 56 years old ( *p* < 0.001), and it was necessary to remove 2.6 cm in length of the canal to reduce the chance of recurrence ( *p* < 0.006). The rate of invasive cancer was 2.7%.

**Conclusion** Cytology was related to a high prevalence to histological lesion (73%) in the diagnosis of CIN2+ in the endocervical disease; older patients presented a higher relationship with histological lesions in the canal disease, and it was necessary to remove an average of 2.6 cm in length of the endocervical canal to avoid the persistence and progression of CIN. The rate of occult neoplasia in the endocervical canal was 2.7%.

## Resumo

**Objetivo** Avaliar o papel da citologia oncótica (CO) no diagnóstico da neoplasia intraepitelial cervical 2 ou maior (NIC2 + ) presente exclusivamente no canal endocervical, as características clínico-epidemiológicas deste tipo de lesão, o comprimento necessário de canal a ser retirado na conização, e a taxa de lesão invasora oculta no canal endocervical.

**Métodos** Estudo transversal, por análise de base de dados, de pacientes com citologia alterada, sem lesão colposcópica visível, submetidas a conização por cirurgia de alta frequência (CAF), para avaliar a associação dos resultados citológicos com o produto histológico da conização, as características epidemiológicas da lesão endocervical, e evolução clínica, utilizando o valor de *p* < 0.05 e intervalo de confiança (IC) de 95%.

**Resultados** Nos 444 casos analisados, a sensibilidade da CO para o diagnóstico de NIC 2+ foi de 75% (IC 95%: 69.8–79.7), a especificidade foi de 40% (IC 95%: 30.2–49.5), e a taxa de prevalência de lesão histológica foi de 73% (IC 95%: 70.1–78.7). Houve maior prevalência de NIC2+ em pacientes com mais de 42 anos de idade e de neoplasia invasora naquelas com mais de 56 anos ( *p* < 0.001), e foi necessário a retirada de 2.6 cm de comprimento de canal para diminuir a taxa de recidiva ( *p* < 0.006). Foi identificada uma taxa de 2.7% de neoplasia invasora.

**Conclusão** A citologia esteve relacionada a uma alta prevalência de lesão (73%) no diagnóstico das NIC2+ na doença endocervical; quanto maior a idade, maior foi a relação da histologia com a citologia de canal, e se fez necessário retirar uma média de 2.6 cm de comprimento de canal para evitar a persistência e a progressão da NIC. A taxa de neoplasia oculta no canal endocervical foi 2.7%.

## Introduction

Cervical intraepithelial neoplasia (CIN) are proliferative lesions with abnormal and atypical maturation of varying degrees, replacing part or all of the thickness of the cervical squamous epithelium, and representing the precursor lesions of cervical cancer, whose diagnosis and treatment allows for a reduction in mortality by this neoplasm. 1 2

Cervical cancer is the third most frequent tumor in the female population, after breast and colon cancer, and the fourth leading cancer-related cause of death in women in Brazil. About 300,000 women each year die from this type of cancer, despite its high possibility of prevention and early treatment. Its incidence is concentrated in the age group from 25 to 59 years old. However, the risk increases significantly in the age group from 45 to 49 years. 1 2

The prevention of cervical cancer consists of early diagnosis, through screening of its precursor lesions, cervical intraepithelial neoplasia (CIN), more specifically high-grade lesions (high-grade squamous intraepithelial lesion [HSIL]/CIN 2,3). 1 2 3 4 5

According to data from the World Health Organization (WHO), 1 99% of high-grade intraepithelial lesions and invasive cancers of the cervix are caused by the human papillomavirus (HPV) and can be detected early through well-organized screening programs. 1 2 3 4 5

In addition to HPV, studies indicate smoking, low intake of vitamins, multiple partners, and early sexual initiation as risk factors for the development of CIN, the precursor to cervical cancer. Added to this, low socio-educational level associated with low population coverage of screening programs for precursor lesions of cervical cancer in health services increase the incidence and mortality from this neoplasm. 1 2 3 The diagnostic methods for these precursor lesions are morphological, such as cervical cytology (Pap smears, Pap tests), colposcopy and histology, or, in most cases, their association. 3 4 5

Because it is a cheap and accessible test, cervical cytology is the preferred method of screening for cervical cancer and its precursor lesions in many countries. Despite its high specificity, it has low sensitivity. Thus, false negative results may be due to inadequate collection, inadequate fixation, or unsatisfactory material. Thus, studies point to the need for the association of another diagnostic method to increase the sensitivity in tracking precursor lesions, such as HPV-DNA research or colposcopy and/or cervical biopsy. 3 4 5

The conventional periodic Pap test, or cervical cytology collected from the cervix, remains the most used strategy for tracking intraepithelial lesions in Brazil. When the patient's result shows changes with a high probability of representing cancer or preinvasive lesions, immediate referral for colposcopy is necessary. 6 7

When CIN is detected in the uterine ectocervix, or in cases that reach the endocervical canal as far as the entire transformation zone can be seen, the treatment is performed by loop electrosurgical procedure (LEEP). 5 6 7 8 However, when the lesion is located entirely in the endocervical canal, or the transformation zone is not fully visible, colposcopy has limitations, and it is necessary to histologically investigate the endocervical canal. 6 7 8

This research aims to verify the role of cytology (Pap smears), collected exclusively from the endocervical canal of the uterine cervix, in women with an abnormal cytology (suggesting a high-grade lesion), with colposcopy disagreements (whose colposcopy does not reveal injury), and submitted to a LEEP procedure for diagnosis and relate the cytology results with those histological results of the conization product, calculating the prevalence of histological lesion in the endocervical canal. In addition, it aims to identify the clinical and epidemiological characteristics of the disease presented exclusively in the endocervical canal, and to evaluate the average length of endocervical canal excised necessary to obtain a conization product with free margins, thus ensuring an appropriate treatment to prevent progression to invasive cancer, and to identify the rate of invasive cancer hidden in these specific cases.

## Methods

This is a cross-sectional study, performed by analyzing a database of patients with abnormal cytological diagnosis of the uterine cervix, suggesting CIN2 + , without visible colposcopic lesion. These patients underwent conization, for diagnosis, to evaluate the association of cytology results with the histological product of the conization, to identify the prevalence of histological lesion in the endocervical canal and the epidemiological characteristics of endocervical disease, and to determine the clinical evolution in these cases, using *p* -value < 0.05 and 95% CI.

The database included patients evaluated at the cervical pathology service of the gynec oncology department at Hospital Erasto Gaertner (HEG), from January 2009 to December 2016. The patients had abnormal Pap smears suggesting cervical HSIL, without visible colposcopic lesion. They underwent a second Pap test, collected exclusively from the endocervical canal, which presented a result of HSIL, atypical squamous cell, cannot rule out high **-** grade squamous intraepithelial lesion (ASC-H), atypical glandular cell (AGC) or adenocarcinoma in situ (AIS), invasive squamous cell carcinoma (SCC) or low-grade squamous intraepithelial lesion (LSIL) and atypical squamous cell of undetermined significance (ASC-US) (according to Bethesda terminology). Additionally, they were submitted to investigation of the endocervical canal using the LEEP procedure, because HPV test is not accessible in the public health system due to its high cost.

The exclusion criteria were all cases that presented a visible colposcopic lesion in the ectocervix and whose diagnosis was made by cervical biopsy.

The data collected from this database were transferred to an Excel spreadsheet (Microsoft Corp., Redmon, WA, USA) and analyzed using the IBM SPSS Statistics for Windows, Version 25.0 software (IBM Corp., Armonk, NY, USA), seeking a confidence interval (CI) greater than 95% and a significance level of 5% ( *p* ≤ 0.05). The qualitative variables were analyzed by the chi-squared test and/or Fisher exact test, with the *p* -value identified. And the quantitative variables were analyzed by the Student *t* -test, and the *p* -value was identified. The sensitivity and specificity of cytology collected exclusively from the endocervical canal were calculated to identify the validity of this method in diagnosing endocervical injury consistent with CIN 2 + .

A waiver of the free and informed consent form was requested, as it is not a study with living beings and whose subject are data from laboratory tests belonging to the database of the aforementioned service.

The research was approved by the research ethics committee (CAAE 61845916.6.0000.0098).

Thus, data of 4,016 patients analyzed in the period, using the inclusion and exclusion criteria, resulted in 444 cases in the sample.

The following variables were analyzed: mean age of patients, menopausal status, cytology result, histopathological result of the conization piece, length of the endocervical canal removed, margins of the conization product, percentage of invasive neoplasia found in the histological conization product, recurrence rate.

## Results

Of the 444 patients evaluated, the mean age was 44 years ± 12 years (95% CI: 43–45/standard error 0.61), with a minimum age of 19 years and a maximum of 89 years. In addition, 32% (143 cases) were in climacteric and 35% of the cases were over 35 years old.

The alterations found in endocervical canal cytology (second cytology) were: HSIL and ASC-H (68%), followed by LSIL (17%), ASCUS (10%), AGC (4%), and, finally, invasion (1%). The prevalence rate of histological lesion in the conization product was 73% (324/444) of the cases with abnormal endocervical cytology, confirming endocervical disease. Of these, 64% (285 cases) were CIN2 + , 27% (120 cases) were cervicitis, 6% (26) were CIN1, and 3% (13) were invasive cancer. Added to this, 42% (175) of the cases revealed glandular extension in the histological conization product.

From the sample of 444 cases, it was possible to verify the margins in 437 patients. The result found was: 88% (383/437) had free margins. Among the 12% of compromised margins (54/437), 46% (25 cases) had compromised endocervical margin, 44% (24 cases) had ectocervical margin, and 9% (5 cases) had both compromised margins.

Regarding the length of the removed canal, the mean was 2.36 cm ± 1.04 cm (95% CI: 2.26–2.46/standard error 0.05). The minimum length of canal removed was 0.5 cm, and the maximum was 5.6 cm. Of all cases, 84% had between 1 to 3.5 cm in length of the endocervical canal removed.

Of the 444 patients, 347 patients had adequate follow-up for recurrence analysis (minimum follow-up time of 18 months), with long-term follow-up being possible. Among these cases, the recurrence rate was 23% (79/347). In relation to margins, 37% (16/43) of committed margins and 21% (63/303) of free margins relapsed, *p* -value = 0.021.

It was found that the mean length of the canal removed in patients with disease recurrence was 2.3 ± 0.9 cm (95% CI: 2.09–2.55), MIN 0.5, MAX 5.3cm, while the mean for cases without recurrence was 2.6 ± 0.8 cm (95% CI: 2.5–2.7, MIN 0.5, MAX 5.6 cm/ *p* -value = 0.006, demonstrating statistical significance.

The mean age of patients without recurrence was 43 years ± 12 (95% CI: 43 ± 12 (CI: 43–45), MIN 20, MAX 89 years, and those with recurrence were: 48 ± 13 (95% CI: 45–51), MIN 23, MAX 83 years/ *p* -value = 0.03, demonstrating statistical significance.

Table 1 shows the relationship between age and the histological result of the endocervical canal lesion, showing a direct relationship between increasing age and the severity of the lesion, with clinical significance.

**Table 1:** Relationship between the result of the pathology report of the conization product and the patient's age

Anatomopathological	Average age
Cervicitis	46 years ± 12 (95% CI: 44–49)
CIN1	38 years ± 11 (95% CI: 47–64)
CIN2	42 years ± 13 (95% CI: 34–43)
CIN3	44 years ± 11 (95% CI: 40–44)
Invasion	56 years ± 13 (95% CI: 42–46)

Abbreviations: CI, confidence interval; CIN, cervical intraepithelial neoplasia.

Table 2 demonstrates the correlation of cytological results collected exclusively from the endocervical canal with the definitive histological result of the piece of endocervical canal obtained from the conization product, demonstrating that cytology with a result suggestive of high-grade lesion has the highest prevalence of similar results in histology, with *p* -value < 0.001, demonstrating statistical significance.

**Table 2:** Relationship between cytology result exclusively from the endocervical canal and the anatomopathological result of the conization product, demonstrating a greater correlation between histology and cytology with the increase in the degree of severity of the lesion/ *p* < 0.001 ( *n* = 444)

	CERVICITIS	CIN1	CIN2	CIN3	INVASION
ASCUS	34% (16/46)	7% (3/46)	33% (15/46)	24% (11/46)	2% (1/46)
LSIL	34% (26/76)	17% (13/76)	40% (30/76)	8% (6/76)	1% (1/76)
HSIL	25% (51/204)	3% (6/204)	41% (84/204)	30% (61/204)	2% (5/204)
ASC-H	19% (19/99)	3% (3/99)	33% (33/99)	42% (42/99)	3% (2/99)
AGC	50% (8/16)	0% (0/16)	25% (4/16)	6% (1/16)	19% (3/16)

Abbreviations: AGC, atypical glandular cell; ASC-H, atypical squamous cells, cannot rule out high-grade squamous intraepithelial lesion; ASCUS, atypical squamous cells of undetermined significance; CIN, cervical intraepithelial neoplasia; HSIL, high-grade squamous intraepithelial lesion; LSIL, low-grade squamous intraepithelial lesion.

The sensitivity and specificity of endocervical canal cytology for diagnosing high grade and/or invasive cervical intraepithelial neoplasia in patients with exclusive endocervical canal lesion can be seen in Table 3 .

**Table 3:** Values related to the sensitivity and specificity of the endocervical cytology to diagnose high-grade squamous intraepithelial lesion and cervix invasive carcinoma

Histological lesion	Sensitivity	Specificity
CIN 2–3	74.3% (95% CI: 69–79)	40.3% (95% CI: 30–50)
Invasion SCC	50% (95% CI: 1.26–88)	90.8% (95% CI: 84.5–95.1)
CIN 2 + *	75% (95% CI: 69.8–79.7)	39.6% (95% CI: 30.2–49.5)

Abbreviations: CI, confidence interval; CIN, cervical intraepithelial neoplasia.

CIN2 + = any lesion greater than CIN 2

The rate of occult invasive neoplasia in the endocervical canal was 2.7% (12/444 cases).

## Discussion

Cervical cancer is the fourth most prevalent cancer diagnosis in women worldwide, and, in our country, it corresponds to the third most common tumor in women and the fourth cause of death from cancer. The incidence and mortality from this type of cancer are related to prevention and screening programs. But to achieve significant results in reducing mortality, knowledge of the clinical and epidemiological characteristics of the disease and the population affected by it is necessary. 1 2 9

The endocervical disease is not so well known, and its diagnostic limitations delay treatment and worsen prognosis. Based on this, the data shown here sought to better delineate the characteristics of endocervical disease.

According to data from this research, endocervical disease was more severe and more prevalent with older age, suggesting that postmenopausal women with positive cytology and absence of colposcopic lesion should not fail to investigate the presence of endocervical canal lesion. In view of the direct relationship found between cases of invasive cancer and advancing age, the use of this method (exclusive cytology of the endocervical canal) in screening for these lesions is justified.

It also concludes that the recurrence rate is higher in women with exclusively endocervical lesions, and that it is necessary to remove a greater depth and length of the endocervical canal to ensure adequate treatment for this specific type of lesion. The prevalence rate of occult invasive cancer in the endocervical canal was 2.7%.

Cytology collected from the endocervical canal was related to a high prevalence of histological lesion in the diagnosis of CIN2 + , justifying its use in the screening of suspected endocervical disease.

Although oncotic cytology is the main test for detecting changes in the cervix, it still has limitations, showing false-negative results. 3 The main factors related to low specificity are due to sampling errors, such as inadequate collection of material, inadequate fixation of the slide, and misinterpretation of the findings. 10

The assessment of cytology quality is related directly to the assessment of the adequacy of samples, established by the Bethesda system, which advocates patient identification, relevant clinical information, technical interpretability, and cell composition. According to the literature, suitability can reach up to 99% in samples in which rapid review of negative smears, rapid prescreening, and automated review is possible. 3

A Brazilian retrospective study found among 97 women with abnormal cytology, 88 (78.6%) also had abnormal histological examination, a result that is in accordance with the accuracy of sensitivity and lesion prevalence obtained in the present study, in which the presence of histological lesion was found in 73% of patients with cytology collected exclusively from the endocervical canal, without visible colposcopic lesion. This brings to debate the need to investigate the endocervical canal in patients with repeatedly altered cytology, due to the high prevalence of precursor lesion, which, when not identified and treated, has a high risk of progression to invasive cancer. 11

According to an assessment performed by CICAN-Bahia/Brazil, which evaluated the accuracy of oncotic cytology, the sensitivity and specificity found were, respectively, 85% and 40%. These values agree with the results presented in this work (75% and 39%, respectively). On the other hand, Nkwabong et al., 12 in their published article, found a sensitivity of 55.5% and a specificity of 75%. 10 12

The literature data corroborate the findings related to relapse and compromised margins. It is notorious that compromising margins increase the chance of relapse. However, the presence of free margins is not a sufficient factor to prevent recurrences. 3 9 13 14 15 16

However, relevant data from the research developed here demonstrate that recurrences occur in a greater proportion in disease located exclusively in the endocervical canal, when compared with the mean recurrence of ectocervical and/or CIN with visible endocervical component. Using as a parameter the literature data, 9 13 15 16 which demonstrate a recurrence rate between 8 and 15% in free margins and above 15% in compromised margins, the data produced here showed a recurrence rate in the free margins of 21%, that is, twice as many lesions located in the ectocervix.

Another piece of data in agreement with this study is in relation to the length of the canal removed. According to Rosa and Lisboa, 14 the greater the length of the excised canal, the lower the recurrence rate. However, the same study highlights that 8% of women, even with free margins, will have residual disease. 14

Carvalho et al., 16 in their literature review, demonstrated that excised endocervical canal lengths smaller than 1.25 cm increase recurrence rates, in agreement with the data produced here, in which, in the presence of a totally endocervical lesion, there would be a need for removal minimum 2.6 cm in length of the endocervical canal, so that the risk of recurrence and/or persistence of the disease is lower.

Another important point to be highlighted in exclusively endocervical disease is that the older the patient, the greater the relationship between the presence of a more severe histological lesion, since patients over 50 years old had a higher risk of presenting invasive carcinoma in the conization specimen, and those over 40 years of presenting a high-grade lesion (CIN 2 + ). In the same line of research, the mean age found in recently published articles was 43, with 5 years for the diagnosis of a precursor lesion, which corroborates the mean age found in this work (44 years). 11 12 14

The relationship between older age and disease severity was also found by a baseline cohort study performed in the Netherlands, which indicates that women over 50 years of age diagnosed with CIN 3 are 7 times more likely to develop cervical cancer. 15

Although the data found in this study were enlightening regarding the role of cervical cytology, the importance of early diagnosis of the disease present in the endocervical canal and the epidemiological characteristics of this specific population, this study presented a limitation: it did not carry out the HPV-DNA. The research does not allow correlating the data with the HPV typing with the severity of the endocervical lesion, which may be an influencing factor in the relationship of cytology data with the histological diagnosis. But it is important to clarify that this limitation is related to issues concerning the cost of this type of test in low-income countries such as Brazil, with this sample having been collected from patients assisted in the Brazilian Public Health System.

## Conclusion

Cytology was related to a high prevalence of histological lesion (73%) in the diagnosis of CIN2+ in the endocervical disease; the older ages present a higher relationship with histological lesions in the canal disease, and it was necessary to remove an average of 2.6 cm in length of the endocervical canal to avoid the persistence and progression of CIN. The rate of occult neoplasia in the endocervical canal was 2.7%.
